# Author Correction: A syndromic surveillance tool to detect anomalous clusters of COVID-19 symptoms in the United States

**DOI:** 10.1038/s41598-021-97597-6

**Published:** 2021-09-03

**Authors:** Amparo Güemes, Soumyajit Ray, Khaled Aboumerhi, Michael R. Desjardins, Anton Kvit, Anne E. Corrigan, Brendan Fries, Timothy Shields, Robert D. Stevens, Frank C. Curriero, Ralph Etienne‑Cummings

**Affiliations:** 1 Department of Electrical and Computer Engineering, Johns Hopkins Whiting School of Engineering, The Johns Hopkins University, 3400 N. Charles Street, 105 Barton Hall, Baltimore, MD 21218 USA; 2 Department of Epidemiology, Spatial Science for Public Health Center, Johns Hopkins Bloomberg School of Public Health, Baltimore, MD 21205 USA; 3 Department of Anesthesiology and Critical Care Medicine, Neurology, Neurosurgery and Radiology, Johns Hopkins University School of Medicine, Baltimore, MD 21205 USA

Correction to: *Scientific Reports* 10.1038/s41598-021-84145-5, published online 25 February 2021

The Supplementary Information file published with this Article contained an error in the spreadsheet, where the data columns were incorrectly rendered.

This error has now been corrected in the Supplementary Information file that accompanies the original Article.

